# Capilloquinol: A Novel Farnesyl Quinol from the Dongsha Atoll Soft Coral *Sinularia capillosa*

**DOI:** 10.3390/md9091469

**Published:** 2011-08-30

**Authors:** Shi-Yie Cheng, Ki-Jhih Huang, Shang-Kwei Wang, Chang-Yih Duh

**Affiliations:** 1 Department of Marine Biotechnology and Resources, National Sun Yat-sen University, Kaohsiung 804, Taiwan; E-Mails: shiyie63@yahoo.com.tw (S.-Y.C.); m965020019@student.nsysu.edu.tw (K.-J.H.); 2 Department of Microbiology, Kaohsiung Medical University, Kaohsiung 807, Taiwan; 3 Centers for Asia-Pacific Ocean Research and Translational Biopharmaceuticals, National Sun Yat-sen University, Kaohsiung 804, Taiwan

**Keywords:** soft coral, *Sinularia capillosa*, farnesyl quinoid, cytotoxicity

## Abstract

Capilloquinol (**1**), possessing an unprecedented farnesyl quinoid skeleton, was isolated from the Dongsha Atoll soft coral *Sinularia capillosa*. The structure of capilloquinol was elucidated by extensive analysis of spectroscopic data. The cytotoxicity and antiviral activity against human cytomegalovirus of **1** was evaluated *in vitro*.

## 1. Introduction

**Figure 1:**
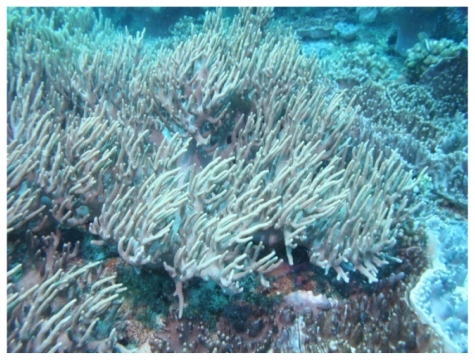
Soft coral *Sinularia capillosa*.

**Figure 2:**
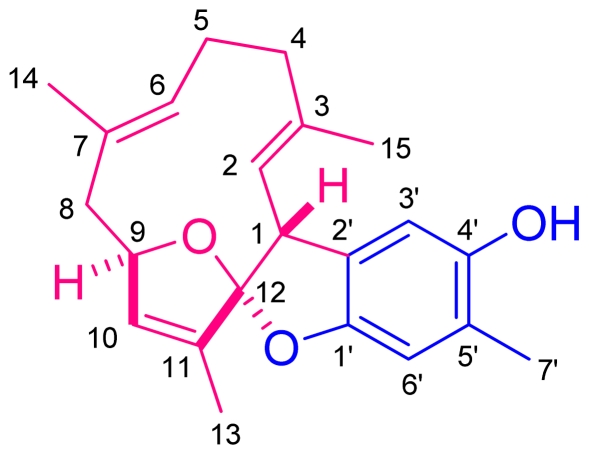
The structure of capilloquinol (**1**).

Numerous farnesyl quinones and quinols [1], possessing a wide range of structural diversity, have been obtained from marine organisms, including sponges [2–8], algae [8,9], ascidians [10–12], and soft corals [13,14]. Many of these mixed C_15_ + C_6_ analogues are of considerable interest due to their cytotoxicity [4,6,7,12], anti-HIV-1 reverse transcriptase activity [4,8,15], and antimicrobial activity [4,6]. As part of a continuing search for bioactive substances from marine invertebrates, chromatographic separation on the acetone extract of the Dongsha Atoll soft coral *S. capillosa* (Figure 1) resulted in the isolation of capilloquinol (**1**) (Figure 2), an unprecedented farnesyl quinoid having novel carbon skeleton. The details of isolation and structural elucidation of metabolite **1** are discussed in this paper. The cytotoxicity against P-388 (mouse lymphocytic leukemia), HT-29 (human colon adenocarcinoma), and A-459 (human lung adenocarcinoma) cancer cell lines as well as antiviral activity against human cytomegalovirus (HCMV) of **1** were evaluated. Meanwhile, the posible biosynthetic pathway for **1** was postulated see below.

## 2. Results and Discussion

The acetone extract of *S. capillosa* was concentrated to a brown gum, which was partitioned with EtOAc and H_2_O. The EtOAc-soluble residue (60 g) was subjected to Si 60 CC using *n*-hexane–EtOAc mixtures of increasing polarity for elution, to yield 40 fractions. Fraction 14 (0.8 g), which eluted with *n*-hexane–EtOAc (2:1), was applied to a RP-18 gravity column and flushed with MeOH–H_2_O (9:1). The ensuing fraction (72 mg) was further purified by RP-18 High-performance liquid chromatography (HPLC) eluted with MeOH–H_2_O (9:1) to yield **1** (2.0 mg, 0.003%).

Capilloquinol (**1**) was obtained as a colorless, viscous oil. The HRESIMS of **1** exhibited a [M + Na]^+^ peak at *m/z* 361.1778, consistent with the molecular formula of C_22_H_26_O_3_, implying ten degrees of unsaturation. Its IR spectrum absorption at 3406 cm^−1^ revealed the presence of hydroxyl group(s). The NMR spectroscopic data (Table 1) of **1** contained resonances for five trisubstituted double bonds [δ_H_ 5.01 (d, *J* = 10.0 Hz, 1H); δ_C_ 137.5 (qC) and 125.7 (CH); δ_H_ 4.86 (br d, *J* = 10.5 Hz, 1H); δ_C_ 130.3 (qC) and 132.2 (CH); δ_H_ 5.97 (s, 1H); δ_C_ 135.9 (qC) and 131.8 (CH); δ_H_ 6.46 (s, 1H); δ_C_ 129.7 (qC) and 112.0 (CH); δ_H_ 6.45 (s, 1H); δ_C_ 152.2 (qC) and 111.7 (CH)] and a tetrasubstituted double bond [δ_C_ 150.8 (qC) and 125.0 (qC)]. The above functionalities accounted for six of the ten degrees of unsaturation, implying a tetracyclic structure for **1**.

**Figure 3:**
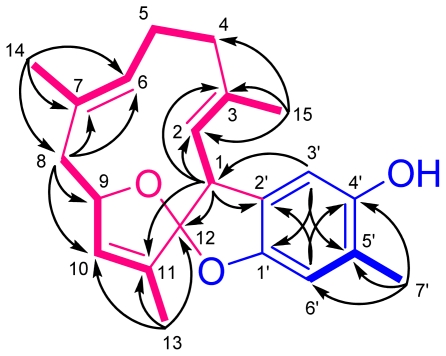
Key ^1^H–^1^H COSY (**—**) and HMBC (→) correlations of **1**.

By interpretation of ^1^H–^1^H and long range COSY correlations (Figure 3), it was possible to establish three partial structures of consecutive proton systems extending from H-1 to Me-15 through H-2, from H_2_-4 to Me-14 through H_2_-5 and H-6, from H_2_-8 to Me-13 through H-9 and H-10. The linkages between C-3 and C-4; C-7 and C-8; C-11 and C-12; were elucidated on the basis of the HMBC correlations (Figure 3) from Me-15 to C-2, C-3, and C-4, from Me-14 to C-6, C-7, and C-8, and from Me-13 to C-10, C-11, and C-12. The HMBC spectrum showed correlations from H-1 to C-2, C-3, C-11, and C-12, proving the linkages from C-1 to C-11 through C-12. Additionally, the crucial HMBC correlations from H-3′ to C-1′ and C-5′, from H-6′ to C-2′ and C-4′, and from Me-7′ to C-4′, C-5′, and C-6′, demonstrated the presence of a 1,2,4,5-tetrasubstituted benzene ring. These HMBC correlations also confirmed the positioning of the oxygen-bearing quaternary carbons at C-1′ and C-4′ [δ_C_ 152.2 (qC) and 150.8 (qC)], and the methyl group at C-5′. Although there were no direct HMBC correlations available, the remaining two degrees of unsaturation indicated that the two oxygen bridges must be present between C-9/C-12 and C-12/C-1′. This assumption was further supported by its NMR spectroscopic data [δ_H_ 5.00 (1H, br s, H-9); δ_C_ 85.3 (CH, C-9) and 125.6 (qC, C-12)] [16] and revealed the presences of a 2,3-dihydrobenzofuran-5-ol moiety and an 13-oxa-bicyclo[8,2,1]tridecane ring. Moreover, the crucial HMBC correlation from H-3′ to C-1 established ring fusion at C-1 and C-12. Therefore, the planar structure of **1** was proposed as shown in Figure 2.

**Figure 4:**
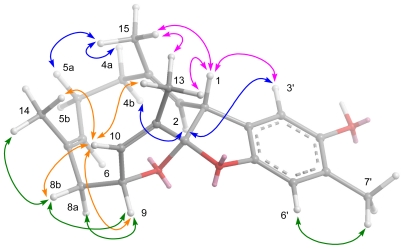
Selected NOESY correlations of **1**.

The geometries of the trisubstituted double bonds were assigned as 2*E* and 6*E* based on the crucial NOESY correlations (Figure 4) between H-1/Me-15, H-4b/H-2, and H-8b/H-6. The key NOE correlations between H-1/Me-13, H-1/Me-15, Me-13/Me-15, Me-15/H-4a, Me-15/H-5a, Me-13/H-10, H-10/Me-14, Me-14/H-8b, and H-8b/H-10 suggested that these protons were oriented on the same side of the macrocyclic ring, while H-2, H-4b, H-5b, H-6, H-8a, and H-9 were oriented on the opposite side. The above-mentioned findings indicated the 1R*, 9R*, and 12R* configurations as depicted in Figure 4. The results, together with other detailed NOESY correlations (Figure 4) of **1**, determined the structure of capillosanol as shown in Figure 1.

**Scheme 1:**
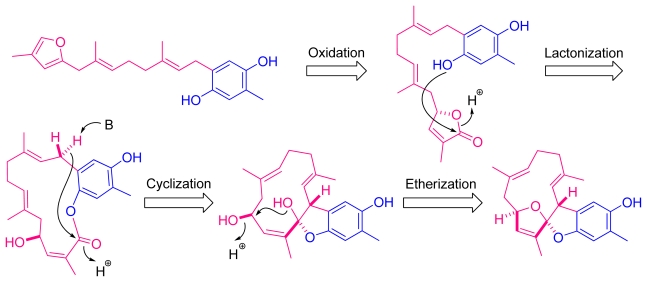
Possible biogenetic pathway for **1**.

It is worthwhile to mention that the framework of **1** may be involved in the possible biosynthesis of a farnesyl quinol [13] through oxidation, lactonization, cyclization, and etherization to result in the formation of capilloquinol (**1**) as depicted in Scheme 1.

Capilloquinol (**1**) was evaluated for cytotoxicity against P-388, A-459, and HT-29 cancer cell lines as well as antiviral activity against human cytomegalovirus. Metabolite **1** displayed cytotoxicity against P-388, with an ED_50_ of 3.8 μg/mL. With the exception of the above finding, **1** did not show cytotoxic against A-459, and HT-29 cancer cell lines, nor was it active against human cytomegalovirus (HCMV).

## 3. Experimental Section

### 3.1. General Experimental Procedures

Optical rotations were determined with a JASCO P1020 digital polarimeter. Ultraviolet (UV) and infrared (IR) spectra were obtained on a JASCO V-650 and JASCO FT/IR-4100 spectrophotometers, respectively. The NMR spectra were recorded on a Varian Unity INOVA 500 FT-NMR spectrometer at 500 MHz for ^1^H and 125 MHz for ^13^C, respectively. Chemical shifts are expressed in δ (ppm) referring to the solvent peaks δ_H_ 3.30 and δ_C_ 49.0 for CD_3_OD, respectively, and coupling constants are expressed in Hz. ESI-MS were recorded by ESI FT-MS on a Bruker APEX II mass spectrometer. Silica gel 60 (Merck, Germany, 230–400 mesh) and LiChroprep RP-18 (Merck, 40–63 μm) were used for column chromatography. Precoated silica gel plates (Merck, Kieselgel 60 F_254_, 0.25 mm) and precoated RP-18 F_254s_ plates (Merck) were used for thin-layer chromatography (TLC) analysis. High-performance liquid chromatography (HPLC) was carried out using a Hitachi L-7100 pump equipped with a Hitachi L-7400 UV detector at 220 nm together with a semi-preparative reversed-phase column (Merck, Hibar LiChrospher RP-18e, 5 μm, 250 × 25 mm).

### 3.2. Animal Material

The soft coral *S. capillosa* was collected by hand using SCUBA along the coast reefs offshore from the Dongsha Atoll in April 2007, at a depth of 8–10 m, and was stored in a freezer at −20 °C for two months until extraction. Identification was kindly verified by Prof. Chang-Feng Dai, Institute of Oceanography, National Taiwan University, Taiwan. A voucher specimen (TS-06) was deposited in the Department of Marine Biotechnology and Resources, National Sun Yat-sen University, Taiwan.

### 3.3. Extraction and Isolation

The acetone extract of *S. capillosa* was concentrated to a brown gum, which was partitioned with EtOAc and H_2_O. The EtOAc-soluble residue (60 g) was subjected to Si 60 CC using *n*-hexane–EtOAc mixtures of increasing polarity for elution, to yield 40 fractions. Fraction 14 (0.8 g) was applied to a RP-18 gravity column to obtain a mixture (72 mg) that was further purified by RP-18 HPLC eluted with MeOH–H_2_O (9:1) to yield **1** (2.0 mg).

The frozen soft coral (2 kg) was chopped into small pieces and extracted exhaustively by maceration with fresh acetone for 24 h at room temperature. The quantity of solvent used for each extraction (2 L) was at least three times the amount of the soft coral material used. The acetone extracts were filtered and concentrated under vacuum to yield a brownish oily residue, which was subsequently partitioned between EtOAc and H_2_O. The resulting EtOAc-soluble residue (60 g) was subjected to column chromatography on silica gel using *n*-hexane with increasing amounts of EtOAc, and finally 100% MeOH as elution, to fractionate roughly 40 fractions on the basis of the ^1^H NMR spectroscopic data and TLC analyses. Fraction 14 (0.8 g) eluted with *n*-hexane–EtOAc (2:1) was subjected to was applied to a RP-18 gravity column to obtain a mixture (72 mg) that was further purified by RP-18 HPLC eluted with MeOH–H_2_O (9:1) to yield **1** (2.0 mg).

Capilloquinol (**1**): colorless, viscous oil; [α]_D_ ^25^ +31 (*c* 0.1, CHCl_3_); IR (KBr) ν_max_ 3406, 3038, 2973, 2928, 17498, 1457, 1422, 1737 cm^−1; 1^H NMR and ^13^C NMR data, see Table 1; ESIMS *m/z* 361 [M + Na]^+^; HRESIMS *m/z* 361.1778 [M + Na]^+^ (calcd. for C_22_H_26_O_3_Na, 361.1780).

### 3.4. Cytotoxicity Assay

Cytotoxicity was determined against P-388 (mouse lymphocytic leukemia), HT-29 (human colon adenocarcinoma), and A-549 (human lung epithelial carcinoma) tumor cells using a modification of the MTT [3-(4,5-dimethylthiazo-2-yl)-2,5-diphenyltetrazolium bromide] colorimetric method. The provision of the P-388 cell line was provided by J. M. Pezzuto, formerly of the Department of Medicinal Chemistry and Pharmacognosy, University of Illinois at Chicago. HT-29 and A-549 cell lines were purchased from the American Type Culture Collection. The experimental details of this assay were carried out according to a previously described procedure [17–19].

### 3.5. Anti-HCMV Assay

To determine the effects of natural product upon HCMV cytopathic effect (CPE), confluent human embryonic lung (HEL) cells grown in 24-well plates were incubated for 1 h in the presence or absence of various concentrations of tested natural product. Then, cells were infected with HCMV at an input of 1000 pfu (plaque forming units) per well of 24-well dish. Antiviral activity was expressed as IC_50_ (50% inhibitory concentration), or compound concentration required to reduce virus induced CPE by 50% after 7 days as compared with the untreated control. To monitor the cell growth upon treating with natural products, an MTT-colorimetric assay was employed [20].

## Figures and Tables

**Table 1:** ^1^H and ^13^C NMR Spectroscopic Data of **1** a.

	^13^C	^1^H
1	46.5 (CH) b	4.31 d (10.0)
2	125.7 (CH)	5.01 d (10.0)
3	137.5 (qC)	
4	40.3 (CH_2_)	a: 2.23 dt (12.0, 3.5) c b: 2.13 dt (12.0, 4.5)
5	26.5 (CH_2_)	a: 2.31 dddd (13.0, 12.0, 10.5, 3.5) b: 2.01 br d (13.0)
6	132.2 (CH)	4.86 br d (10.5)
7	130.3 (qC)	
8	45.1 (CH_2_)	a: 2.51 dd (14.0, 4.5) b: 2.28 br d (14.0)
9	85.3 (CH)	5.00 s
10	131.8 (CH)	5.97 s
11	135.9 (qC)	
12	125.6 (qC)	
13	12.0 (CH_3_)	1.85 br s
14	18.9 (CH_3_)	1.47 s
15	16.0 (CH_3_)	1.57 s
1′	152.2 (qC)	
2′	129.7 (qC)	
3′	112.0 (CH)	6.45 s
4′	150.8 (qC)	
5′	125.0 (qC)	
6′	111.7 (CH)	6.46 s
7′	16.7 (CH_3_)	2.13 s

a Spectra were measured in CD_3_OD (^1^H, 500 MHz and ^13^C, 125 MHz);

b Multiplicities are deduced by HSQC and DEPT experiments;

c *J* values (in Hz) are in parentheses.
